# Deep graph contrastive learning model for drug-drug interaction prediction

**DOI:** 10.1371/journal.pone.0304798

**Published:** 2024-06-17

**Authors:** Zhenyu Jiang, Zhi Gong, Xiaopeng Dai, Hongyan Zhang, Pingjian Ding, Cong Shen

**Affiliations:** 1 College of Information and Intelligence, Hunan Agricultural University, Changsha, China; 2 School of Computer Science and Engineering, Hunan University of Information Technology, Changsha, China; 3 Key Laboratory of Intelligent Perception and Computing, Hunan University of Information Technology, Changsha, China; 4 School of Computer Science, University of South China, Hengyang, China; 5 School of Physical and Mathematical Sciences, Nanyang Technological University, Singapore, Singapore; The University of Alabama in Huntsville, UNITED STATES

## Abstract

Drug-drug interaction (DDI) is the combined effects of multiple drugs taken together, which can either enhance or reduce each other’s efficacy. Thus, drug interaction analysis plays an important role in improving treatment effectiveness and patient safety. It has become a new challenge to use computational methods to accelerate drug interaction time and reduce its cost-effectiveness. The existing methods often do not fully explore the relationship between the structural information and the functional information of drug molecules, resulting in low prediction accuracy for drug interactions, poor generalization, and other issues. In this paper, we propose a novel method, which is a deep graph contrastive learning model for drug-drug interaction prediction (DeepGCL for brevity). DeepGCL incorporates a contrastive learning component to enhance the consistency of information between different views (molecular structure and interaction network), which means that the DeepGCL model predicts drug interactions by integrating molecular structure features and interaction network topology features. Experimental results show that DeepGCL achieves better performance than other methods in all datasets. Moreover, we conducted many experiments to analyze the necessity of each component of the model and the robustness of the model, which also showed promising results. The source code of DeepGCL is freely available at https://github.com/jzysj/DeepGCL.

## Introduction

Drug–drug interaction (DDI) refers to the phenomenon that occurs when two or more drugs are taken together, resulting in adverse effects on an organism [1, 2]. Thus, how to accurately identify drug-drug interactions has become an important research content. Traditional methods which used in drug-drug interaction identification are mainly based on experimental assays and clinical reports [3]. However, this process would be costly and time-consuming, especially for identifying drug-drug interactions from a large drug space. Computational methods (in silico [4–6]) can be used as an effective and fast alternative to alleviate this problem. Among these methods usually focus on learning single drug properties and lack effective integration of multiple sources of drug-related information, which ultimately limits the predictive capabilities of the model. Therefore, it has become an important research direction in the field of drug discovery to propose an effective and fast calculation method for drug-drug interaction prediction.

In recent years, accumulated research findings have demonstrated promising results in computational-based drug-drug interactions (DDIs) prediction. These achievements are primarily attributed to the rapid advancements in drug molecular property prediction [7–10]. These methods for predicting DDIs can be broadly categorized into two groups: Structure-based methods and network-based methods. Firstly, structure-based methods mainly consider the entire drug as a graph or sequence. For example, some researchers consider atoms as nodes and bonds between atoms as edges, then use a graph neural network (GNN) to learn the representation of each drug [11–17]. Additionally, some models use SMILES (Simplified Molecular Input Line Entry System) [18] as the input for sequence models (including GRU [19], LSTM [20], and Transformer [21]), then predict the DDIs. In these methods, drugs are treated as independent individuals, and the representation is learned from the drug molecular structure and then transported to the classifier through some aggregating operations. Next, another important method for predicting DDIs is the network-based method. In this kind of method, the authors mainly consider the drug as a node, and then consider the interaction or similarity between drugs as an edge to form a large network, and then use the traditional network science method or the graph neural network method to predict the unknown interaction of drug molecules [22–24]. Although these methods have achieved good performance, they still have some limitations. Firstly, the structure-based methods assume that drugs with similar features will behave similarly in the DDIs, however, there may be a lower similarity between interacting drugs. Meanwhile, the performance of the network-based methods relies on the quality of the interaction network, and it is time-consuming and difficult to build large-scale high-quality networks. Second, the drug molecular graph and the drug interaction network contain mutually irreplaceable pharmacological properties, which are very important for predicting DDIs. The drug molecular graph contains information about the drug functional groups that determine the chemical and physical properties of the drug. The topological information between drugs is contained in the interaction network, which contains some specific functions of some drugs. Although these methods obtain great performance in some specific tasks, they focus only on single-view learning and ignore the mutual complementarity of information among multi-view.

Existing research has demonstrated the effectiveness of building models to predict DDIs from multiple perspectives, primarily by aggregating multi-source information, including drug structure information, network topological information, and more [25–27]. For example, MUFFIN [28] has aggregated molecular structure information and drug topology information to predict DDIs. DSN-DDI [29] has utilized both local and global representation learning modules, which can learn drug substructures from individual drugs (intra-view) and drug pairs (inter-view) simultaneously. m2vec [22] has combined drug target networks with SMILES information and then used graph autoencoders to learn the final representation of drugs. The success of these methods confirmed the advantages of predicting DDIs from the multi-view. However, these methods prioritize leveraging multi-view data to improve drug representation, without considering the balance and consistency of multi-source information, and cannot effectively utilize the structure-level and network-level information. Contrastive learning is often used to maximize the mutual information between multiple perspectives. Thus, researchers have used the contrastive learning component to balance and integrate molecular structure information and interaction network information [30]. Moving forward, if the drug pair can be directly regarded as a whole, the representation vector can be learned at the level of the drug pair, which can be used to model training and DDI prediction, it may provide a new perspective for DDI prediction.

In this paper, we introduce a novel Deep Graph Contrastive Learning model (DeepGCL) for drug-drug interaction prediction. DeepGCL leverages graph contrastive learning to combine both molecular structure features and network topological features. Firstly, DeepGCL constructs the molecular structure graph for each drug and employs a graph convolutional network (GCN) to learn the structural features of the drugs. Then, DeepGCL constructs a subgraph for each drug pair and utilizes GCN to learn the topological features of each drug pair. To better choose a pooling operation, it is important to emphasize that we utilize a virtual node to aggregate the node features of the entire subgraph. Next, the graph contrastive learning model is used to combine the features of drug structure and network topology. Finally, the structural and topological features of the learned drug pairs are integrated for drug-drug interaction prediction. Experimental results demonstrate that DeepGCL achieves the best performance across three real-world datasets. In our study, we performed an ablation analysis which unequivocally demonstrated the essential role of graph contrastive learning in integrating information from various perspectives. Meanwhile, we also conducted experiments to assess the robustness of the DeepGCL model.

## Method

### DeepGCL framework

**Fig 1:**
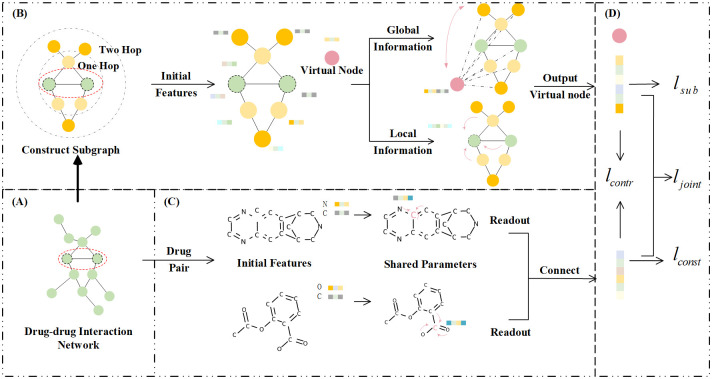
Overview of DeepGCL. (A) Drug-drug interaction networks: This component illustrates the network of interactions between drugs. (B) Topology information learning module: This module extracts common H-Hop neighbor nodes for drug pairs to form a subgraph. Subsequently, the subgraph is passed through GCN to generate a global representation for the drug pair. (C) Structural information learning module: This module employs GCN with shared parameters to acquire representations for drugs within drug pairs. (D) Graph contrastive learning module and prediction module: In this module, graph contrastive learning and cross-entropy loss are employed to regulate the model’s iteration and predict the interaction probability between input drug pairs.

The overview of the DeepGCL is shown in Fig 1. DeepGCL is decomposed into three parts: (1) Topology information learning module (Fig 1B). This module mainly uses a graph convolutional network to learn the representation of each node in a local subgraph. (2) Structural information learning module (Fig 1C). This module utilizes a graph convolutional network to learn the representation of each drug, and drugs of the drug pair share parameters during the learning process. (3) Graph contrastive learning module and prediction module (Fig 1D). This module mainly uses graph contrastive learning and cross-entropy loss to constrain the model iteration and predict the probability of interaction between input drug pairs. Firstly, all drugs form an interconnected network in which the drugs represent nodes, and the edges represent interactions between the drugs (Fig 1A). Then, we sample the common H-Hop neighbor nodes from the drug interaction network for any input drug pair to construct a subgraph. Meanwhile, we introduce a virtual node to learn the global features of the subgraph, which is connected to all nodes within the subgraph. Additionally, we utilize the internal structural information of drug molecules to construct molecular graphs. Furthermore, to balance the information between the molecular and subgraphs of the drug pair, we incorporate a graph contrastive learning module that optimizes the model by ensuring consistency in the representation between the molecular and subgraphs. Finally, the prediction of drug-drug interactions combines the molecular structure and topology information of drug pairs.

### Subgraph construction and representation learning

In recent years, the use of graph neural networks for analyzing networked graph data has garnered considerable attention. For example, Scorpius constructs a knowledge graph to evaluate the correlation between drugs and diseases [31]. However, dealing with large-scale networks can pose significant computational challenges. Therefore, there’s increasing interest in extracting topological information from subgraphs. For instance, DisenCite utilizes L hops neighbors from the paper relationships network to learn topological information [32]. Inspired by this, DeepGCL learns topological relationships among drugs within a specific neighborhood by selecting H-Hop neighboring nodes and edges to construct subgraphs. This approach enables DeepGCL to concentrate on learning local drug pair features from subgraphs without the need to train the entire drug interaction network. If two drugs lack shared neighbors, the subgraph consists of only those two drugs, otherwise, shared neighbors are used to form subgraphs.

Given a drug interaction network graph *G*_*I*_ = (*V*, *E*), V denotes the set of nodes in the graph, where *v*_*i*_ ∈ *V* denotes the *i*-th drug in the drug interaction network. *E* denotes the set of edges in the graph, where each edge represents an interaction between drugs. *N*_*v*_(*h*) denotes the set of H-Hop neighbors of node *v*. For any sample (*v*_*i*_, *v*_*j*_), we sample the first-order neighbor for the node and obtain the second-order neighbors from the first-order neighbors, and so on. Then we can obtain $N_{v_{i}}\left(h\right)$ and $N_{v_{j}}\left(h\right)$, we choose the common neighbors of two drugs to form the drug interaction subgraph denoted as $N_{v_{i}}\left(h\right)\cap N_{v_{j}}\left(h\right)$ as the nodes that form the subgraph *G*_*sub*_.

We obtain the subgraph *G*_*sub*_ = (*A*, *X*) using the aforementioned sampling method. Here, $A\in R^{n\times n}$ is the adjacency matrix, $X\in R^{n\times d}$ is the node feature matrix, and *d* is the dimension of node features. It is essential to highlight that we initialize the node features using 166-dimensional Morgan fingerprints [33]. For learning drug interaction network subgraph features, we employ a variant of Graph Convolutional Networks [34]. Unlike traditional graph convolutional networks that aggregate information from neighboring nodes, our approach focuses on extracting global information from the entire graph using virtual nodes. These virtual nodes are connected to all other nodes in the graph, facilitating direct communication between higher-order neighboring nodes to capture global graph features. The virtual node feature vector is initialized as the average of the other node feature vectors. After adding the virtual node, the subgraph $G_{sub}^{\prime }=\left(A_{t},X\right)$ based on node feature matrix $X\in R^{(n+1)\times d}$, where $A_{t}\in R^{\left(n+1)(n+1\right)}$ is the adjacency of subgraph and *n* + 1 is the number of nodes in the subgraph. Then, the node features are updated by a multilayer graph convolutional neural network, defined as follows:

$$\begin{array}{c} Z_{t}^{\left(l\right)}=ReLU({\tilde{D}}_{t}^{-\frac{1}{2}}{\tilde{A}}_{t}{\tilde{D}}_{t}^{-\frac{1}{2}}Z_{t}^{\left(l-1\right)}W_{t}^{\left(l\right)}) \end{array}$$ (1)

where $W_{t}^{\left(l\right)}$ is the *l*-th layer weight matrix, and *ReLU* is the activation function. ${\tilde{A}}_{t}=A_{t}+I_{t}$ and $\tilde{D}$ is the diagonal matrix of ${\tilde{A}}_{t}$. Finally, we extract the features of virtual nodes as the global features of subgraphs, as follows:

$$\begin{array}{c} Z_{T}=z_{virtual}^{l} \end{array}$$ (2)

where $z_{virtual}^{l}$ denotes the feature representation of the virtual nodes in the *l* level. Next, binary classification using *MLP*, as follows:

$$\begin{array}{c} p_{sub}=MLP\left(Z_{T}\right) \end{array}$$ (3)

Then, calculating cross-entropy loss *l*_*sub*_ using the predicted probability distribution *p*^*sub*^ from embedding *Z*_*T*_, as follows:

$$\begin{array}{c} l_{sub}=-\frac{1}{N}\sum_{i=1}^{N}[y_{i}\;\text{log}\left(p_{i}^{sub}\right)+\left(1-y_{i}\right)\;\text{log}\left(1-p_{i}^{sub}\right)] \end{array}$$ (4)

where *y*_*i*_ is the truth label (0 or 1) and $p_{i}^{sub}$ is the predicted probability of sample *i*.

### Molecular structure representation

This section employs GCN with shared parameters to learn drug molecular structural representations. Specifically, we use the open-source software RDKit [35] to transform the drug SMILES into molecular graphs. In these molecular graphs, the nodes and edges correspond to atoms and bonds within the molecule, respectively. Given a molecular graph defined as *G*_*m*_ = (*A*, *X*), where $A\in R^{m\times m}$ denotes the symmetric adjacency matrix with *m* nodes, $X\in R^{m\times h}$ is the node feature matrix, *h* is the dimension of the matrix. Each node represents an atom, and *A*_*i*,*j*_ = 1 indicates the existence of a covalent bond between nodes *i*, *j*, and 0 otherwise. For molecular structure feature initialization, we follow the approach used in DeepDDS [12], which utilizes DeepChem [36] to calculate five pieces of information: atomic symbols and the number of adjacent atoms.

In each layer of the GCN, every node aggregates information from itself and its surrounding neighboring nodes to acquire higher-level feature representations. For the input drug molecular graph (*A*_*f*_, *X*), the output $Z_{cf}\in R^{m\times h^{\prime }}$ can be defined as below:

$$\begin{array}{c} Z_{cf}^{\left(k+1\right)}=ReLU({\tilde{D}}_{f}^{-\frac{1}{2}}{\tilde{A}}_{f}{\tilde{D}}_{f}^{-\frac{1}{2}}Z_{cf}^{\left(k\right)}W_{c}^{\left(k+1\right)}) \end{array}$$ (5)

where $W_{c}\in R^{h^{\prime }\times h^{\prime }}$ represents the shared weight parameter, *h*′ is the dimensionality of output features and is not affected by the number of nodes. ${\tilde{A}}_{f}=A_{f}+I_{f}$ and $\tilde{D}$ is the diagonal matrix of ${\tilde{A}}_{f}$ and $Z_{cf}^{0}=X$ is the initial feature matrix, and $Z_{cf}^{k+1}$ denotes the feature representation of the nodes in the k+1 layer. In order to learn similar information between drug pairs, for the molecular graph (*A*_*t*_, *X*), the weight parameter *W*_*c*_ is shared at each layer in both GCNs, as follows:

$$\begin{array}{c} Z_{ct}^{\left(k+1\right)}=ReLU({\tilde{D}}_{t}^{-\frac{1}{2}}{\tilde{A}}_{t}{\tilde{D}}_{t}^{-\frac{1}{2}}Z_{ct}^{\left(k\right)}W_{c}^{\left(k+1\right)}) \end{array}$$ (6)

where $Z_{ct}^{0}=X$ is the initial feature matrix, and $Z_{ct}^{k+1}$ denotes the feature representation of the nodes in the k+1 level. Then the graph-level representation of each molecular graph extracted is concatenated to obtain the embedding *Z*_*C*_ of the drug pair, as follows:

$$\begin{array}{c} Z_{C}=Readout\left(Z_{ct}\right)\|Readout\left(Z_{cf}\right) \end{array}$$ (7)

where ∥ is the concatenate operation. In DeepGCL, the READOUT function performs max pooling. Next, MLP is used to classify tasks as follows:

$$\begin{array}{c} p^{const}=MLP\left(Z_{C}\right) \end{array}$$ (8)

The cross-entropy loss *l*_*const*_, as follows:

$$\begin{array}{c} l_{const}=-\frac{1}{N}\sum_{i=1}^{N}[y_{i}\;\text{log}\left(p_{i}^{const}\right)+\left(1-y_{i}\right)\;\text{log}\left(1-p_{i}^{const}\right)] \end{array}$$ (9)

### Graph contrastive learning

Due to the scarcity of labeled data, unsupervised learning has been widely applied in the fields of few-shot learning [37], recommendation systems [38, 39], and natural language processing [40]. Among them, KGNN combines graph neural networks and kernel-based networks to effectively utilize both labeled and unlabeled graphs [41]. Additionally, graph contrastive learning represents one of the most advanced unsupervised learning methods, with successful applications in various tasks, including node classification, graph classification, and drug discovery [42–44]. Some graph contrastive learning models commonly use a contrastive learning framework based on graph augmentation, such as edge perturbation [45], node deletion [46], and attribute augmentation [47] to form a contrastive view. However, methods that perturb the structure of the input graph to obtain different contrasting views may introduce noise, potentially affecting the model’s performance. Therefore, DSGC [48] constructs contrasting views in different spaces and fits the advantage of each by graph contrastive learning. Inspired by this, both drug molecule graphs and subgraphs contain rich information about drug interactions, and we can leverage contrastive learning to combine the two to obtain better embeddings.

For drug molecule graphs and subgraphs, after the nonlinear mapping introduced above, we obtain their low-dimensional representations. The representation of the molecular graph contains information on multiple functional groups, while the subgraph contains local topological information within the interaction network. The consistency of the representation vectors can be maximized by adding a contrastive learning component.

Specifically, we feed a batch of samples of size N into the model, and a total of 2N embeddings are obtained through the model. Negative pairs are formed between samples from the same batch, and positive pairs are formed between two views produced by the same sample. Then the loss can be expressed as the distance between a positive sample and the remaining K negative samples. Thus, for sample *i*, we have the following self-supervised loss.

$$\begin{array}{c} l_{contr}^{i}=-\text{log}(\frac{e^{sim(Z_{T_{i}},Z_{C_{i}})/\tau }}{\sum_{k=1}^{N}(e^{sim(Z_{T_{i}},Z_{C_{k}})/\tau }+e^{sim(Z_{T_{i}},Z_{C_{i}})/\tau })}) \end{array}$$ (10)

where *τ* is the temperature coefficient, $Z_{C_{i}}$ and $Z_{T_{i}}$ are the molecular graph embedding and subgraph embedding of sample *i*, respectively. *sim*(*Z*_*T*_, *Z*_*C*_) denotes the cosine similarity between two vectors. The total contrastive loss of all samples is defined as follows:

$$\begin{array}{c} l_{contr}=-\frac{1}{N}\sum_{i=1}^{N}l_{contr}^{i} \end{array}$$ (11)

### Drug-drug interaction prediction

For each sample, the framework generates a molecular graph representation and a subgraph representation of the drug pair through its two channels. Subsequently, the relationship between the two embeddings is modeled through various operations to obtain the final representation of the drug pair. Finally, we utilize this representation as input to MLP and evaluate the predicted interaction score, as follows:

$$\begin{array}{c} p_{ct}=MLP(\|(Z_{C}+Z_{T},Z_{C}⊙Z_{T},Z_{C},Z_{T})) \end{array}$$ (12)

where ⊙ is the inner product between vectors. The binary vector *p*_*ct*_ represents the associated probabilities of drug pairs. Label is 1 for interacting drug pairs and 0 otherwise. Subsequently, we calculate the cross-entropy loss, denoted as *l*_*joint*_, to measure the dissimilarity between predicted probabilities and the true labels, as follows:

$$\begin{array}{c} l_{\text{joint}}=-\frac{1}{N}\sum_{i=1}^{N}[y_{i}\;\text{log}\left(p_{i}^{ct}\right)+\left(1-y_{i}\right)\;\text{log}\left(1-p_{i}^{ct}\right)] \end{array}$$ (13)

From this, DeepGCL’s overall loss function can be described as follows:

$$\begin{array}{c} l_{total}=\alpha l_{contr}+\beta l_{const}+\lambda l_{sub}+\eta l_{joint} \end{array}$$ (14)

where *l*_*const*_ measures the discrepancy between the model’s predictions of molecular structural information and the ground truth, while *l*_*sub*_ quantifies the difference between the model’s predictions of the topological information of drug-pair interaction subgraphs and the ground truth. *l*_*joint*_ represents the disparity between model predictions and the true situation after aggregating molecular structure information and subgraph topology information. *l*_*contr*_ represents the graph contrastive learning loss for unsupervised tasks. The *α*, *β*, λ, and *η* are the coefficients of different losses.

## Experiments

In this section, we demonstrate the performance of the model on two real-world datasets to test the effectiveness of the model in the task of predicting adverse drug reaction classification to answer the following three questions:

- Q1: How does DeepGCL perform in real-world datasets compared to other models?
- Q2: Does integrating information from drug molecular graphs and subgraphs improve the performance of the model?
- Q3: After adding the contrastive learning component, can the model further improve the learning ability of the model?

### Dataset and baseline

DeepGCL is a binary classification model that focuses on detecting drug interactions, which utilizes three real-world datasets (BioSNAP [49], AdverseDDI [50], and DrugBank [51]) to verify performance. After preprocessing drug SMILES strings with RDKit, we excluded drugs lacking SMILES representations and corresponding molecular data. In this model, the positive-to-negative sample ratio is 1:1. Details are shown in Table 1.

**Table 1:** Dataset statistics.

	BioSNAP	AdverseDDI	DrugBank
Drugs	1306	388	569
Positive DDI labels	41010	12288	37243
Negative Labels	41010	12288	37243

To verify the validity of DeepGCL and answer Q1, we compare it with two types of models, which are graph neural network models and network embedding models. The graph neural network models include CSGNN [52], DeepDDI [53], DeepDDS [12] and CASTER [8]. Among these, DeepDDI and DeepDDS use drug structure information to learn drug representation. CSGNN proposes that a hybrid multi-Hop neighborhood aggregator will be incorporated to capture the interrelationships of indirect neighbors in molecular interaction networks. CASTER considers the functional substructure of the drug, uses a self-encoder to learn the chemical structure data, and increases the interpretability of the model by adding a dictionary learning module. The network embedding model includes Deepwalk [54], Line [55], node2vec [56], SDNE [57], and struc2vec [58]. Deepwalk preserves the similarity between neighboring nodes and Line further preserves the similarity of nodes that have common neighbors. node2vec improves the random wandering strategy and enriches the contextual information of the nodes. SDNE is a semi-supervised learning method that uses self-encoders to simultaneously optimize the similarity of the nodes’ higher-order neighbors and learn the local and global features of the nodes. struc2vec focuses on the spatial structure features of nodes in the network, considering the similarity of nodes in the local topology. NNPS [59] constructs initial features by amalgamating information concerning drug molecular side effects and drug-protein interactions. Subsequently, NNPS employs neural networks to compute the probabilities associated with adverse drug reactions for given drug combinations.

### Experimental setting

To evaluate our model more comprehensively, we randomly split the dataset into a training set, a validation set, and a test set using an 8:1:1 ratio. For each experiment, we randomly split the dataset 5 times. All comparison models were set according to the parameters in the original paper. Referring to the DeepDDS [12], we train two shared parameter 3-layer GCNs for learning drug molecular graphs with dimensions {78, 156, 128}, and train drug interaction network subgraphs with encoder 3-layer GCNs with dimensions {166, 332, 128}. The optimizer is Adam and the dropout rate is {0.2, 0.5, 0.8}. For joint training, we set *α* = 0.1, *β* = 1, λ = 1, *η* = 1. We use the area under the ROC curve (AUC), F1 score (F1), and area under the precision recall curve (AUPR) as metrics to evaluate the model. The training process encompassed a total of 100 epochs.

### Experimental results

In Table 2, we provide the mean and standard deviation of performance metrics for DeepGCL and various baseline models on three real-world datasets. Superior results are highlighted in bold text. DeepGCL consistently exhibits strong performance across all datasets. This observation not only supports the effectiveness of our approach but also provides validation for the research question Q1.

**Table 2:** Comparative evaluation (mean ± std). Best performance in each metric is shown in bold font.

Model	BioSNAP			AdverseDDI			DrugBank		
	AUC	AUPR	F1	AUC	AUPR	F1	AUC	AUPR	F1
CSGNN	0.956_0.000_	0.946_0.000_	0.892_0.006_	0.946_0.000_	0.939_0.000_	0.826_0.000_	0.928_0.002_	0.916_0.003_	0.838_0.006_
DeepDDI	0.949_0.001_	0.946_0.001_	0.881_0.003_	0.948_0.001_	0.949_0.001_	0.877_0.001_	0.959_0.002_	0.957_0.000_	0.893_0.007_
DeepDDS	0.961_0.001_	0.959_0.002_	0.902_0.003_	0.959_0.002_	0.957_0.003_	0.892_0.004_	0.932_0.009_	0.926_0.012_	0.857_0.012_
CASTER	0.915_0.006_	0.888_0.026_	0.845_0.007_	0.906_0.006_	0.898_0.009_	0.835_0.005_	0.861_0.016_	0.857_0.029_	0.788_0.027_
Deepwalk	0.878_0.005_	0.858_0.004_	0.732_0.025_	0.880_0.009_	0.864_0.011_	0.766_0.036_	0.849_0.005_	0.840_0.005_	0.771_0.012_
Line	0.871_0.004_	0.868_0.007_	0.786_0.015_	0.933_0.002_	0.925_0.006_	0.863_0.015_	0.905_0.001_	0.900_0.002_	0.824_0.003_
struc2vec	0.875_0.004_	0.851_0.002_	0.793_0.031_	0.925_0.005_	0.907_0.011_	0.840_0.046_	0.773_0.067_	0.781_0.062_	0.697_0.053_
node2vec	0.813_0.002_	0.805_0.003_	0.719_0.016_	0.854_0.012_	0.822_0.024_	0.809_0.009_	0.847_0.007_	0.821_0.006_	0.690_0.086_
SDNE	0.850_0.005_	0.842_0.006_	0.748_0.020_	0.917_0.005_	0.894_0.008_	0.815_0.025_	0.852_0.004_	0.853_0.004_	0.760_0.010_
NNPS	0.830_0.019_	0.822_0.019_	0.761_0.023_	0.942_0.004_	0.939_0.007_	0.867_0.007_	0.861_0.016_	0.857_0.029_	0.788_0.027_
DeepGCL	**0.974**_0.002_	**0.970**_0.003_	**0.926**_0.004_	**0.965**_0.002_	**0.963**_0.003_	**0.907**_0.004_	**0.983**_0.000_	**0.982**_0.000_	**0.927**_0.004_

As demonstrated in Table 2, network embedding models such as Line and SDNE exhibit performance levels comparable to that of CASTER. This observation underscores the significance of drug topological information within the drug interaction network, placing it on equal importance with the molecular structure information predicted by DDI. While DeepDDS outperforms DeepDDI in the BioSNAP and AdverseDDI datasets, DeepDDI achieves superior performance in the DrugBank dataset. Both models rely solely on molecular structure information. DeepDDS is based on neural network architecture and highlights the potential of neural network models in DDI prediction. It’s noteworthy to emphasize that DeepDDI’s exceptional performance can be attributed to its integration of additional drug databases for drug structure similarity calculations. This incorporation of additional data sources broadens DeepDDI’s scope, allowing it to incorporate a more comprehensive range of drug-related information compared to other models. Among the baselines, CSGNN consistently shows stable performance across all three datasets. This observation suggests that the model’s approach of enhancing communication among higher-order neighbor nodes contributes to its predictive abilities. Comparatively, DeepGCL uses virtual nodes to connect all nodes in the subgraph, and higher-order neighbors communicate through virtual nodes as intermediaries during message passing. DeepGCL outperforms all other compared models. DeepGCL aggregates drug molecular structure and drug topology information to make up for the limitations of single-molecule graph learning.

We have incorporated an array of evaluation metrics, including Mean Average Precision (MAP), Mean Reciprocal Ranking (MRR), and HIT@K metrics. DeepGCL consistently demonstrates competitive performance across these diverse evaluation criteria, as evident in the experimental results presented in S1 Table. These metrics are particularly relevant in the context of drug discovery, where the emphasis is often on identifying the most promising drug candidates for further experimentation. DeepGCL showed reliable performance, indicating its ability to identify potentially interacting drug pairs. In practical drug recommendation scenarios, the top-ranked drug pairs are of paramount importance, and our model’s proficiency in this regard further underscores its utility in drug discovery.

Furthermore, we analyzed the training time of the model on various datasets to evaluate its computational efficiency. As shown in S2 Table, DeepGCL exhibits advantages in computational efficiency compared to several models, notably NNPS and DeepDDI. This superiority can be attributed to the effectiveness of graph neural networks in learning features from graph-structured data. DeepGCL focuses on molecular structure and network topology to enhance drug interaction prediction accuracy, which consequently affects computational efficiency. However, it remains competitive in both model prediction accuracy and computational efficiency.

### Ablation study

To further investigate the necessity and effectiveness of each component of the DeepGCL model and address questions Q2 and Q3, we designed the following variants of DeepGCL for experiments on three datasets. Each variant was trained five times independently, and the mean and standard deviation were calculated five times.

DeepGCL without molecular structure learning (DeepGCL w/o molecular) learns drug interaction information only from drug interaction subgraphs to make predictions about drug pairs.

DeepGCL without subgraph learning (DeepGCL w/o subgraph) learns only the embedding representation of the drug from the molecular graph as the representation vector of the drug pair.

DeepGCL without contrastive learning (DeepGCL w/o contrastive) trains the target based on supervised signals, and the acquired drug pair embeddings are used for downstream binary classification.

**Fig 2:**
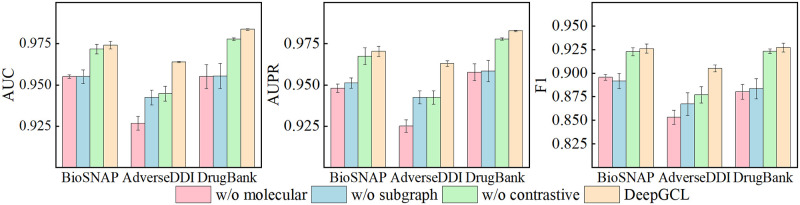
Comparative performance of DeepGCL and its variants on multiple evaluation metrics.

Fig 2 shows the results of DeepGCL and its variants for AUC, AUPR, and F1 scores. It’s evident that removing any component from DeepGCL results in weaker performance compared to the model before removal. These results demonstrate the necessity of the existence of each component in the DeepGCL model. In the BioSNAP and DrugBank datasets, the model’s performance is not significantly improved after adding contrastive learning, as observed in the AdverseDDI dataset. This disparity can be attributed to the fact that BioSNAP and DrugBank already contain rich drug interaction information. Consequently, even without the use of contrastive learning, DeepGCL achieves excellent performance by effectively integrating drug structure and interaction information. However, it’s worth noting that the addition of contrastive learning still resulted in performance improvements, albeit to a lesser extent. This indicates that it can effectively improve the predictive power of the model by integrating information from multiple drug perspectives. By incorporating the contrastive learning component, the model’s two encoders can glean more profound insights into the interplay between drug molecules. Consequently, the model’s learning capacity can be further enhanced. In conclusion, each component of the DeepGCL model is necessary and effective.

### Robustness analysis

**Fig 3:**
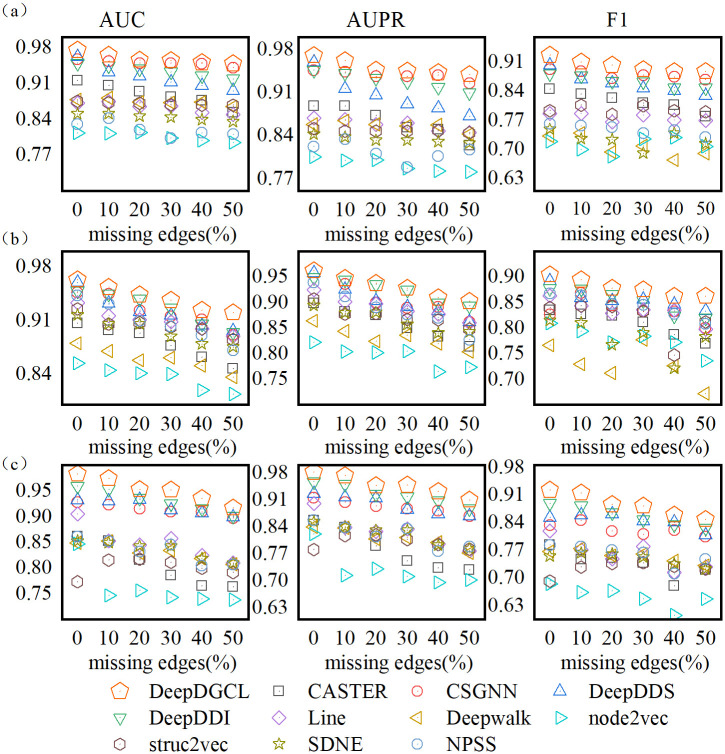
Metrics for models in edge attack scenarios. (a) Performance in BioSNAP dataset, (b) Performance in AdverseDDI dataset, (c) Performance in DrugBank dataset.

Existing deep learning models are susceptible to interference from noise. Here, to verify the robustness of the model, we randomly remove 10%, 20%, 30%, 40%, 50% of known associations. The results are shown in Fig 3. As the corresponding proportion of known associations was removed, all models showed a downward trend, but DeepGCL still performed best in all scenarios. Among graph neural network models, DeepDDS, and DeepDDI perform poorly in the process of removing edges. This can be due to data sparsity problems. These methods begin with drug similarity, learn drug embedding by drug molecular structure information, and assume that similar drugs will have similar performance in DDIs. In comparison, CSGNN shows reliable robustness after removing some edges. The possible reason for this result is that CSGNN uses deep mix-Hop graph neural networks to capture higher-order neighbors to alleviate the problem of data sparsity. In the AdverseDDI dataset, Deepwalk and node2vec degrade performance rapidly as the edges are removed. The above model assumes that nodes with common neighbors will be more similar, and this assumption is easily influenced by noise. In conclusion, DeepGCL can fit the respective advantages of molecular graphs and interaction networks to improve the robustness of the model.

### Cold start experiments

In Drug-Drug Interaction (DDI) prediction tasks, traditional K-fold cross-validation (CV) can inadvertently introduce information overlap between training and testing sets, potentially inflating results. To address this challenge, we employ two distinct cold start scenarios [60]: drug-wise CV and pairwise CV. In drug-wise CV, our objective is to predict interactions between known drugs and unknown drugs. In pairwise CV, the goal is to predict interactions exclusively between unknown drugs. We categorize input drugs into two groups: drugs for training (*drugs*_*train*_) and cold-start drugs (*drugs*_*cold*_) lacking known interactions in the training set. This categorization results in three distinct DDI subsets: *DDI*_*train*_ (for known drugs), *DDI*_*drugwise*_ (for interactions between cold-start and known drugs), and *DDI*_*pairwise*_ (for interactions between cold-start drugs). Subsequently, we utilize logistic regression classifiers trained on *DDI*_*train*_ to make predictions in both drug-wise and pairwise scenarios. The results are presented in Table 3. Remarkably, All the employed models show a noticeable decline in performance across both scenarios when compared to traditional CV. Attributable to DeepGCL’s ability to learn drug representations from both molecular graphs and interaction networks, it exhibits robust performance across two distinct scenarios.

**Table 3:** Assessment of DeepGCL and competitive methods under the drug-wise and pairwise settings. The best score is in bold.

Model		BioSNAP			AdverseDDI			DrugBank		
		AUC	AUPR	F1	AUC	AUPR	F1	AUC	AUPR	F1
CSGNN	drug-wise CV	0.902	0.895	0.8215	0.922	0.906	0.860	0.835	0.826	0.746
	pairwise CV	0.899	0.893	0.816	0.911	0.889	0.842	0.831	0.819	0.736
DeepDDI	drug-wise CV	0.897	0.881	0.823	0.893	0.875	0.818	0.906	0.891	0.831
	pairwise CV	0.888	0.868	0.809	0.876	0.848	0.797	0.898	0.878	0.821
DeepDDS	drug-wise CV	0.892	0.870	0.828	0.923	0.900	0.865	0.894	0.873	0.820
	pairwise CV	0.891	0.867	0.827	0.920	0.891	0.864	0.897	0.877	0.822
CASTER	drug-wise CV	0.827	0.803	0.741	0.882	0.846	0.822	0.811	0.782	0.782
	pairwis CV	0.830	0.805	0.741	0.876	0.841	0.811	0.805	0.774	0.730
Deepwalk	drug-wise CV	0.854	0.835	0.786	0.874	0.854	0.799	0.859	0.842	0.782
	pairwise CV	0.839	0.823	0.770	0.834	0.810	0.750	0.835	0.813	0.754
Line	drug-wise CV	0.865	0.849	0.788	0.915	0.900	0.843	0.873	0.861	0.796
	pairwise CV	0.846	0.829	0.766	0.872	0.844	0.797	0.839	0.824	0.762
struc2vec	drug-wise CV	0.875	0.851	0.808	0.913	0.892	0.845	0.843	0.830	0.763
	pairwise CV	0.859	0.835	0.788	0.879	0.844	0.813	0.802	0.788	0.720
node2vec	drug-wise CV	0.790	0.752	0.728	0.836	0.790	0.775	0.830	0.801	0.754
	pairwise CV	0.765	0.727	0.704	0.781	0.727	0.720	0.801	0.765	0.725
SDNE	drug-wise CV	0.725	0.691	0.661	0.788	0.751	0.713	0.671	0.650	0.619
	pairwise CV	0.679	0.644	0.603	0.691	0.647	0.592	0.578	0.560	0.537
NNPS	drug-wise CV	0.803	0.773	0.724	0.886	0.852	0.818	0.759	0.759	0.777
	pairwise CV	0.770	0.750	0.688	0.855	0.821	0.779	0.746	0.746	0.678
DeepGCL	drug-wise CV	**0.956**	**0.946**	**0.900**	**0.950**	**0.946**	**0.881**	**0.923**	**0.926**	**0.844**
	pairwise CV	**0.955**	**0.945**	**0.900**	**0.952**	**0.949**	**0.883**	**0.913**	**0.918**	**0.831**

### Visualization

**Fig 4:**
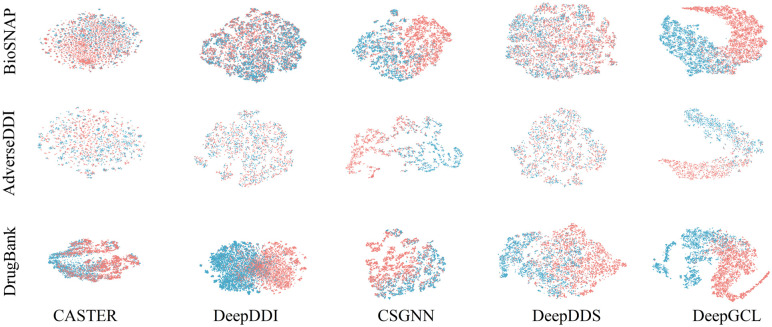
Visualization of DDI network. Red points indicate drug pairs without interactions and green points indicate drug pairs with interactions.

In this section, we analyze the drug pair features learned by DeepGCL. To intuitively observe the relationships between features, we employ dimensionality reduction methods to visualize them as points in a two-dimensional space. Dimensionality reduction methods mainly include linear and nonlinear approaches [61]. Linear LRPER [62] and the nonlinear method T-SNE [63] are two popular dimensionality reduction methods. Given T-SNE’s advantage in preserving local structure, we opt for T-SNE as our dimensionality reduction tool. Since DeepGCL is a deep learning model, we only compare DeepGCL with other deep learning models, including DeepDDS, CSGNN, DeepDDI, and CASTER. As shown in Fig 4, the DeepGCL can well distinguish between drug pairs with (green) and without (red) interactions. In the BioSNAP dataset, we chose the contour coefficients as an indicator to measure the quality of the drug pair representations, and the contour coefficients of DeepGCL, DeepDDS, CSGNN, DeepDDI, CASTER were 0.3246, 0.2085, 0.1973, 0.0188, 0.1944. This indicates that DGCL can better extract the molecular representation of drug pairs.

### Parameter analysis

**Fig 5:**
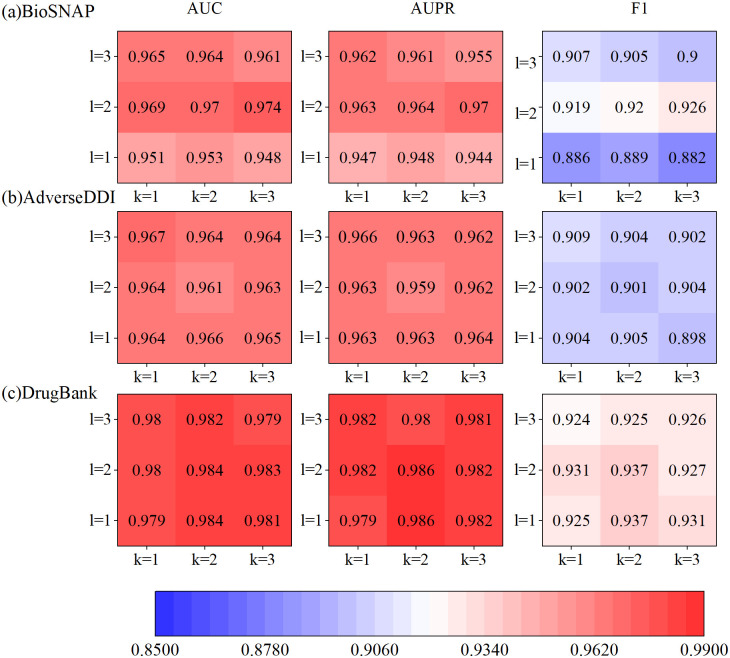
Effect of convolutional neural networks of different depths. (a) Performance in BioSNAP dataset, (b) Performance in AdverseDDI dataset, (c) Performance in DrugBank dataset.

DeepGCL uses GCN to learn semantic information about the graph, where the network depth is crucial for the final learning quality. To verify the effect of different depths of the network on the performance of the model, we build networks of different depths for experiments. The experimental results are shown in Fig 5, where the horizontal axis coordinates correspond to the depth of the learned drug molecular graph network and the vertical axis corresponds to the depth of the learned drug interaction graph network, the darker the color of the module corresponds to the better model performance.

In the BioSNAP dataset, the combination of l = 2 and k = 3 respectively works best, and the combination of k = 1, and l = 3 in the AdverseDDI dataset works best. In the DrugBank dataset, the combination of l = 2 and k = 2 performs best. By evaluating the performance of various combinations, we ultimately chose l = 2 and k = 3.

DeepGCL constructs subgraphs from shared H-Hop drug neighbors to learn drug topology within the DDI network. Increasing H includes more nodes in the subgraph, enhancing topological understanding. In DeepGCL, we select H from the set 1,2 since computational limits. S1 Fig shows that including 2-Hop neighbors enhances performance on AdverseDDI and BioSNAP. In contrast, DrugBank performs optimally with only 1-Hop neighbors due to its higher node degree, where a larger subgraph might introduce noise, potentially counteracting the benefits. In the concluding model, the parameter H is set to 2 for the BioSNAP and AdverseDDI datasets, whereas it is set to 1 for the DrugBank dataset.

## Conclusion

We present DeepGCL, a novel deep graph contrastive learning framework that integrates drug interaction network topology and molecular structure information. DeepGCL constructs subgraphs from shared H-Hop neighboring nodes in the Drug-Drug Interaction (DDI) network and employs GCN to obtain representations for drug molecular graphs and subgraphs. DeepGCL introduces a key graph contrastive learning component to enhance the consistency of embeddings across various perspectives. DeepGCL consistently demonstrates competitive performance across various metrics. When the model is applied to larger datasets, it learns additional topological information from subgraphs, leading to performance improvements. However, this comes with increased computational complexity. In the future, we’ll focus on efficient methods [64] for learning subgraph features, balancing computational efficiency and model performance on large-scale data to improve scalability. In summary, DeepGCL advances drug-drug interaction prediction and maintains a competitive edge in drug interaction research while providing valuable insights.

## Supporting information

S1 Fig The experiment assesses the impact of varying H-Hop neighbors on DeepGCL’s performance across multiple datasets. (TIF)

S1 Table Performance comparison of DeepGCL and competitive methods based on evaluation metrics MRR, MAP, and HIT@K. The best score is in bold.
(DOCX)

S2 Table Training time of models on different datasets (Seconds). (DOCX)
