# Supplementary figures and images for: Deep graph contrastive learning model for drug-drug interaction prediction
Source: PLoS One. 2024 Jun 17;19(6):e0304798. doi: 10.1371/journal.pone.0304798 (PMC11182529; doi:10.1371/journal.pone.0304798)

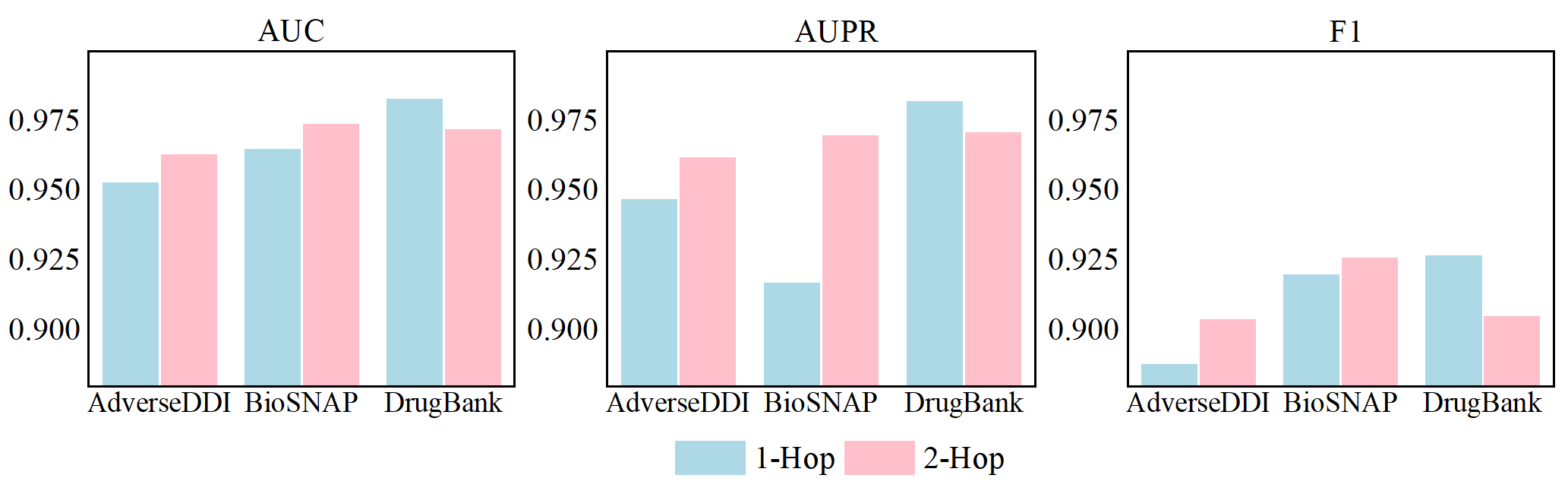

Supplement: S1 Fig — (TIF) [file pone.0304798.s001.tif]
