# Supplementary material for: Deep graph contrastive learning model for drug-drug interaction prediction
Source: PLoS One. 2024 Jun 17;19(6):e0304798. doi: 10.1371/journal.pone.0304798 (PMC11182529; doi:10.1371/journal.pone.0304798)
Supplement: S1 Table — The best score is in bold. (DOCX) [file pone.0304798.s002.docx]

Table S1: Performance comparison of DeepGCL and competitive methods based on evaluation metrics MRR, MAP, and HIT@K. The best score is in **bold**.

| **Dataset** | **Method** | **MAP** | **MRR** | **HIT@1** | **HIT@3** | **HIT@10** | **HIT@100** |
| --- | --- | --- | --- | --- | --- | --- | --- |
| BioSNAP | CSGNN | 0.810 | 0.066 | 0.011 | 0.087 | 0.138 | 0.478 |
|  | DeepDDI | 0.859 | 0.068 | 0.023 | 0.085 | 0.148 | 0.435 |
|  | DeepDDS | 0.875 | 0.041 | 0.001 | 0.051 | 0.090 | 0.445 |
|  | CASTER | 0.752 | 0.024 | 0.008 | 0.020 | 0.043 | 0.221 |
|  | Deepwalk | 0.775 | 0.038 | 0.004 | 0.032 | 0.105 | 0.420 |
|  | Line | 0.823 | 0.026 | 0.001 | 0.011 | 0.055 | 0.334 |
|  | struc2vec | 0.663 | 0.028 | 0.008 | 0.024 | 0.050 | 0.279 |
|  | node2vec | 0.756 | 0.011 | 0.000 | 0.003 | 0.015 | 0.182 |
|  | SDNE | 0.776 | 0.055 | 0.037 | 0.046 | 0.092 | 0.307 |
|  | DeepGCL | **0.907** | **0.178** | **0.103** | **0.212** | **0.301** | **0.711** |
| AdverseDDI | CSGNN | 0.872 | 0.139 | 0.007 | 0.163 | 0.434 | 0.859 |
|  | DeepDDI | 0.879 | 0.135 | 0.054 | 0.142 | 0.301 | 0.709 |
|  | DeepDDS | 0.882 | 0.171 | 0.060 | 0.168 | 0.447 | 0.867 |
|  | CASTER | 0.819 | 0.069 | 0.016 | 0.058 | 0.150 | 0.655 |
|  | Deepwalk | 0.829 | 0.073 | 0.036 | 0.069 | 0.137 | 0.656 |
|  | Line | 0.813 | 0.169 | 0.091 | 0.162 | 0.355 | 0.774 |
|  | struc2vec | 0.822 | 0.028 | 0.008 | 0.024 | 0.050 | 0.279 |
|  | node2vec | 0.682 | 0.044 | 0.012 | 0.028 | 0.083 | 0.513 |
|  | SDNE | 0.780 | 0.055 | 0.037 | 0.046 | 0.092 | 0.307 |
|  | DeepGCL | **0.907** | **0.219** | **0.109** | **0.171** | **0.585** | **0.875** |
| DrugBank | CSGNN | 0.844 | 0.062 | 0.022 | 0.079 | 0.160 | 0.385 |
|  | DeepDDI | 0.895 | 0.123 | 0.077 | 0.124 | 0.208 | 0.557 |
|  | DeepDDS | 0.856 | 0.053 | 0.016 | 0.036 | 0.133 | 0.466 |
|  | CASTER | 0.773 | 0.097 | 0.077 | 0.084 | 0.131 | 0.360 |
|  | Deepwalk | 0.775 | 0.024 | 0.007 | 0.012 | 0.038 | 0.287 |
|  | Line | 0.822 | 0.054 | 0.018 | 0.065 | 0.081 | 0.410 |
|  | struc2vec | 0.706 | 0.044 | 0.016 | 0.043 | 0.075 | 0.306 |
|  | node2vec | 0.756 | 0.011 | 0.003 | 0.009 | 0.015 | 0.102 |
|  | SDNE | 0.766 | 0.032 | 0.005 | 0.040 | 0.070 | 0.286 |
|  | DeepGCL | **0.934** | **0.391** | **0.336** | **0.336** | **0.607** | **0.879** |
