# Supplementary material for: Deep graph contrastive learning model for drug-drug interaction prediction
Source: PLoS One. 2024 Jun 17;19(6):e0304798. doi: 10.1371/journal.pone.0304798 (PMC11182529; doi:10.1371/journal.pone.0304798)
Supplement: S2 Table — (DOCX) [file pone.0304798.s003.docx]

Table S2: Training Time of Models on Different Datasets (Seconds).

|  | **BioSNAP** | **AdverseDDI** | **DrugBank** |
| --- | --- | --- | --- |
| CSGNN | 72.5 | 20.7 | 51.8 |
| DeepDDI | 9119.3 | 2768 | 4034 |
| DeepDDS | 43.7 | 13.0 | 37.8 |
| CASTER | 112.8 | 58.4 | 97.5 |
| Deepwalk | 40.1 | 11.9 | 22.1 |
| Line | 1075.4 | 1068.4 | 994.6 |
| struc2vec | 330.2 | 55.8 | 120.3 |
| node2vec | 508.0 | 110.6 | 485.3 |
| SDNE | 36.7 | 26.7 | 28.3 |
| NNPS | 314.0 | 345.3 | 296.9 |
| DeepGCL | 68.4 | 22.1 | 75.4 |
